# Item-Wise Interindividual Brain-Behavior Correlation in Task Neuroimaging Analysis

**DOI:** 10.3389/fnins.2018.00817

**Published:** 2018-11-06

**Authors:** Xinlin Zhou, Mengyi Li, Hantao Zhou, Leinian Li, Jiaxin Cui

**Affiliations:** ^1^State Key Laboratory of Cognitive Neuroscience and Learning, IDG/McGovern Institute for Brain Research, Beijing Normal University, Beijing, China; ^2^Beijing Advanced Innovation Center for Future Education, Beijing Normal University, Beijing, China; ^3^Siegler Center for Innovative Learning, Beijing Normal University, Beijing, China; ^4^The Second High School Attached to Beijing Normal University, Beijing, China

**Keywords:** brain-behavior correlation, item-wise interindividual brain-behavior correlation, functional magnetic resonance imaging, cognitive neuroscience, mathematical problem-solving

## Abstract

Brain-behavior correlations are commonly used to explore the associations between the brain and human behavior in cognitive neuroscience studies. There are many critics of the correlation approach, however. Most problems associated with correlation approaches originate in the weak statistical power of traditional correlation procedures (i.e., the mean-wise interindividual brain-behavior correlation). This paper proposes a new correlation procedure, the item-wise interindividual brain-behavior correlation, which enhances statistical power via testing the significance of small correlation coefficients from trials against zero rather than simply pursuing the highest correlation coefficient. The item-wise and mean-wise correlation were compared in simulations and an fMRI experiment on mathematical problem-solving. Simulations show that the item-wise correlation relative to the mean-wise correlation results in higher *t-*values when signal-to-noise ratio is equal to or larger than 6%. Item-wise correlation displayed more voxels with significant brain-behavior correlation than did mean-wise correlation. Analyses with item-wise (rather than mean-wise) correlation showed significant brain-behavior correlation at the threshold of *p* < 0.05 corrected. Cross validation showed that odd- and even-ordered trials have greater stability in terms of the item-wise correlation (*r* = 0.918) than the mean-wise correlation (*r* = 0.686). The simulations and example analyses altogether demonstrate the effectiveness of the proposed correlation procedure for task neuroimaging studies.

## Introduction

Neuroimaging researchers often use brain-behavior correlations to explore the association between the brain and human behavior. There are many critics of this approach, however. Problems seemingly inherent to correlation analysis include lack of correction for multiple comparisons (Kriegeskorte and Mur, 2012; Rousselet and Pernet, 2012) and confounding factors in the correlation (Lazic, 2010). Indeed, false correlations have been published in many journals (Vul et al., 2009a,b; Lazic, 2010; Rousselet and Pernet, 2012; Vul and Pashler, 2012). There have been initial attempts to address these problems (Wilcox, 2005; Pernet et al., 2012; Rousselet and Pernet, 2012), but much work remains to be done. The weak statistical power of the routine mean-based interindividual brain-behavior correlation approach is particularly problematic. Previous fMRI analyses of individual differences typically have very little power to detect all but the most powerful correlational effects (Yarkoni, 2009; Yarkoni and Braver, 2010).

This paper introduces a new correlation approach designed for enhanced statistical power. Traditional correlation involves first separately computing the mean values of all items (i.e., trials) on behavioral and brain responses for a participant, then performing correlation across the participants. The traditional correlation typically performed on mean values is referred to in this paper as “mean-wise correlation.” For example, consider 28 participants each of whom finishes 60 trials in an fMRI scanner. Following the procedure for traditional correlation, there are 28 mean values (e.g., reaction time, RT) and 28 brain maps with each voxel of each brain map involving the mean of brain activation from the 60 trials for all participants. The behavioral data (e.g., RT) correlate with the brain activation of each voxel across the 28 participants.

The new correlation approach is a two-step process. A correlation across participants is first performed on the behavioral measure and brain response for an item (i.e., a trial), then the *r-*values are tested via *t*-test or analysis of variance (ANOVA). The proposed correlation approach is performed on an item, so it is referred to here as the “item-wise correlation.” If the item-wise correlation is applied to the example above, there are 60 *r-*values which can be tested with a one-sample *t*-test against 0. The proposed analysis can be performed on a weak correlation due to the abundance of noise for a given item. The *t*-test or ANOVA can greatly enhance the statistical power in this scenario, possibly due to the *r-*values having removed much of the noise.

The traditional mean-wise and novel item-wise interindividual brain-behavior correlations are introduced below. Simulations based on the different signal-to-noise ratios of these two correlation approaches are then described to contrast their respective statistical power. Our fMRI study on mathematical problem-solving is discussed, as well as the application of item-wise and mean-wise correlations to reflect brain-behavior correlations. Cross-validation analysis further demonstrates the statistical stability of item-wise and mean-wise correlations. To conclude the paper, the interindividual correlation is also compared against the intraindividual correlation. For the example mentioned above (28 participants who finish 60 trials in an fMRI scanner), the interindividual brain-behavior correlation is computed first as the correlation between the behavioral score and brain activation for each item across all 28 participants, then a random effect test is run on all the 60 correlation coefficients against zero. The intraindividual correlation involves first computing the correlation between the behavioral score and brain activation for each participant across all 60 trials, followed by a random effect test on the 28 correlation coefficients against zero.

## Item-Based and Mean-Based Interindividual Brain-Behavior Correlations

Most previous research on this subject center around mean-based interindividual brain-behavior correlation, in which the behavioral response and brain response averaged from each participant are correlated across participants. The Pearson correlation is commonly applied to correlation analysis, though outliers can easily expand or dilute the correlations (e.g., Vul et al., 2009a,b; Pernet et al., 2012). The *t*-test formula for the Pearson correlation in this context is as follows:

(1) $$t=\frac{\sqrt{n-2}}{\sqrt{1-r^{2}}}r$$

Cohen provided rules of thumb to characterize effect sizes as small, medium, or large; in the social sciences, *r* = 0.10 generally means small, 0.30 means medium, and 0.50 means a large effect size (Cohen, 1988, 1992) while *r-*values larger than 0.5 are extremely rare in most areas of psychology (Yarkoni and Braver, 2010). The typical effects across broad domains of psychology and medicine range from 0.1 to 0.3 (Meyer et al., 2001). Previous fMRI studies on emotion, personality, and social cognition have shown high brain-behavior correlation values (Vul et al., 2009a).

Magnetic resonance images or functional MRI studies are typically conducted with 30 participants. According to Formula (1), the *t-*value for the large effect size (*r* = 0.50) in 30 cases is still small – only *t*(28) = 3.06 with a corresponding *p-*value of 0.0048. The statistical power is not appropriate under a lenient but acceptable primary threshold (*p* < 0.001) in neuroimaging data analysis (Woo et al., 2014; Carter et al., 2016).

For any effect size (e.g., *r* = 0.50), the sample size can be determined according to the *p-*value. We determined the sample size for our threshold in three steps. First, the *t-*values were calculated for different sample sizes according to Formula (1); *r* was set to 0.50. Second, the Excel TDIST function was used to generate a table for the Student’s T-Distribution based on the *t-*values and different sample sizes (e.g., from 1 to 100). Finally, the sample size was determined according to the *p-*value in the table. For *p* < 0.001, the number of participants for *r* = 0.50 should be at least 40: *t*(38) = 3.56, *p* = 0.0009. For the threshold of *p* < 0.05 corrected, 0.05 should be divided by the number of brain voxels in the 3D whole-brain image by Bonferroni correction method.

The 3D whole-brain volume was set to 61 × 73 × 61 with reference to previously published fMRI studies. The number of brain voxels was 53,462. The *p-*value was determined to be 9.35 × 10^-7^ after correction and the sample size was 86 as per the Student’s T-Distribution table. That is, *t*(84) = 5.29, *p* = 9.32 × 10^-7^. It is difficult to obtain an ideal statistical result without a large number of participants. The item-wise interindividual brain-behavior correlation process involves pursuing high statistical power and statistical stability though the correlation coefficients may be very small. This type of correlation is performed at the item level; an “item” is a trial or a problem. When 28 participants finish 60 mathematical word problems in an fMRI scanner, there are 60 items or trials. First, the brain response of each item is modeled similarly to the first-level analysis in the item-wise brain activation analysis introduced by Bedny et al. (2007). Next, the correlation (e.g., Pearson approach) is computed for each item across participants. Finally, the *r-*values from all items are tested against a fixed number (e.g., 0) or compared to one another. The formula for the typical single-sample *t*-test is as follows:

(2) $$t=\frac{\sqrt{n}(\bar{X}-{\text{μ}}_{0})}{\text{SD}}$$

where the *t-*value depends on sample size (i.e., the number of items), mean of *r-*values (X) and standard deviation of *r-*values (SD). μ_0_is 0 when examining whether brain and behavior are associated. The sample size has similar effect on Formulas (1) and (2). The *r-*value may be small for each item in the item-wise correlation; the *t-*value in Formula (2) could be very large when SD is very small. Thus, the item-wise correlation relative to the mean-wise correlation exacts greater statistical power by relying on possibly weak but stable correlations at the item level.

The one-sample sign test can be applied if the *r-*values for all items are not normally distributed. The *r-*values are first transformed into 0 if they are below the fixed number (e.g., 0), and into 1 if they are above the fixed number. A binomial distribution test can then be applied to compute the *z* score:

(3) $$\text{z}=\frac{K-nP}{\sqrt{nP(1-P)}}$$

where *n* is the sample size (i.e., number of items), *K* is the number of the *r-*values below the fixed number, and *P* is the probability of *r-*value above the fixed number (e.g., *P* = 0.5).

## Simulations for Item-Wise and Mean-Wise Correlations

We compared the two correlation approaches by simulation, as discussed below. All simulations were performed in MATLAB (R2014a, The MathWorks, Inc.). The simulations were first performed on random data (i.e., without any signal) with standard normal distribution. The mean is around 0 and SD is around 1.

**FIGURE 1:**
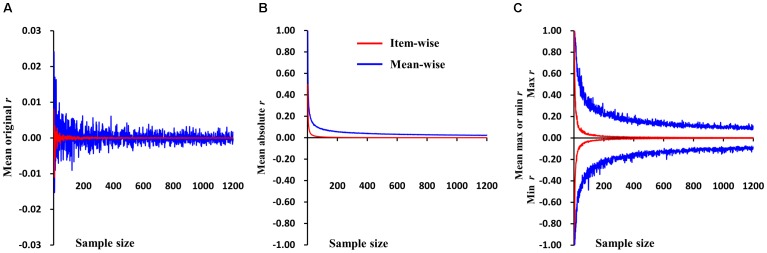
Simulations of item-wise and mean-wise interindividual brain-behavior correlations on same random data (i.e., without any signal). **(A)** Mean r-values for 1,000 samples of each size in item- and mean-wise correlation. **(B)** Item-wise correlation relative to mean-wise correlation with smaller absolute r-values for 1,000 samples at each size. **(C)** Item-wise correlation also results in smaller absolute maximum or minimum r-value.

The simulations were first performed on random values for two variables, then subjected to Pearson correlation. The sample size ranged from 2 to 1,200 with a step of 1. There were 1,000 samples for each sample size. The mean *r-*values for the 1,000 samples of each sample size in item- and mean-wise correlation are reported in Figure 1A. The data are random, so the mean *r-*value is very close to 0 in the item- and mean-wise correlations. The mean *r-*value for item-wise correlation is -0.00001; for mean-wise correlation it is 0.00014. Item-wise correlation relative to mean-wise correlation leads to smaller absolute *r-*values for 1,000 samples at each sample size (from 2 to 1,200), generally *p* < 2.95 × 10^-125^ (Figure 1B). The item-wise correlation also results in smaller absolute maximum or minimum *r-*values (Figure 1C). The *t-*values for each of the three types of *r-*values does not differ between the two correlation approaches.

We performed another simulation on data involving signals. The signal-to-noise ratio in this simulation ranged from 0.01 to 1 with a step of 0.01. Again, there were 1,000 samples for each ratio. The sample size was assumed as 100 participants who finished 100 items. The *x(100,100), y(100,100), and s(100,100)* is represented a 100-by-100 array of pseudorandom values. The *s(100,100)* value was multiplied by the signal-to-noise ratio from 1% to 100%, resulting in signal (S). The signal was added into *x*(100, 100) and *y*(100, 100) as follows:

X=x(100, 100)+S

Y=y(100, 100)+S

Thus, X represents one type of score (e.g., RT) of 100 participants finishing 100 items and Y represents another type of score (e.g., brain activation) of 100 participants finishing 100 items.

**FIGURE 2:**
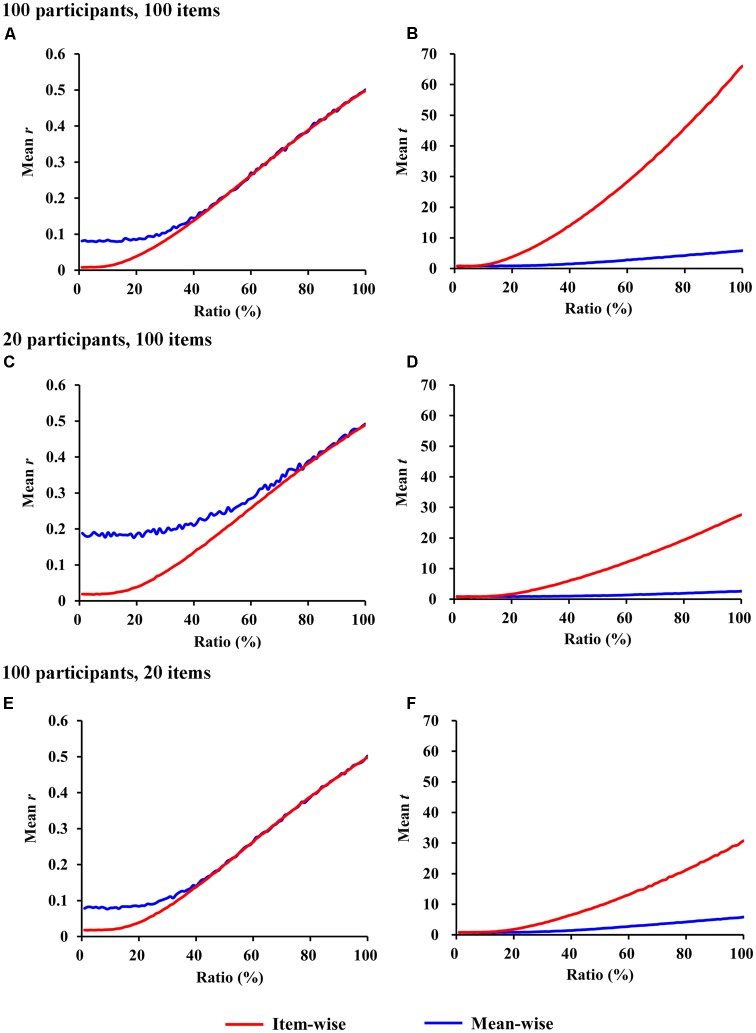
Simulations of item-wise and mean-wise interindividual behavior-brain correlations on same data with signal-to-noise ratios from 1 to 100% (1,000 samples for each ratio). **(A,C,E**) r-Values for item-wise and mean-wise correlation. **(B,D,F)** t-Values for item-wise and mean-wise correlation.

The *r-*values for item-wise correlation are smaller than the *r-*values for mean-wise correlation when the ratio is less than 40% (*p* < 0.05). No differences were observed beyond this value between the two correlations (Figure 2A). For ratios less than 5% (*p* > 0.05), the *t-*values we calculated do not differ between item-wise and mean-wise correlation. When the ratio is 6%, significance emerges at *p <* 0.05. As the ratio increases, the significance becomes more salient. When the ratio is 100%, the mean *t-*value for item-wise correlation is 10 times that for mean-wise correlation (Figure 2B).

The number of participants and the number of items always differ in a study. The simulations for fewer participants and more items (e.g., 20 participants and 100 items) and for more participants and fewer items (e.g., 100 participants and 20 items) are also presented in Figures 2C–F. The simulations resulted in similar patterns to those in the previous simulation (100 participants and 100 items).

The simulations effectively prove the validity of item-wise interindividual behavior-and-brain correlation analysis. We next directly compared the two approaches in a neuroimaging experiment on mathematical problem-solving. We sought to verify that the item-wise correlation reveals neural markers of cognition with sufficient statistical power.

## Example Description and Data Preprocessing

### Participants

Twenty-eight healthy, right-handed university students (11 male, 17 female) were recruited from Beijing Normal University, China. The average age of the subjects was 21.5 years-old within a range from 18.7 to 26.6 years old at the time of this study. They self-reported normal or corrected-to-normal eyesight, normal hearing, and lack of neurological or psychiatric illness. Subjects had no brain abnormality on their T1-weighted high-resolution magnetic resonance images (MRI) as determined by a neuroradiologist. Informed written consent was obtained from each subject after the procedures were fully explained. The experiment was approved by the State Key Laboratory of Cognitive Neuroscience and Learning at Beijing Normal University. Participants were compensated 100 RMB for their time.

### Materials

Sixty mathematical word problems were presented to the participants; participants were required to compute the exact answer to each problem. The problems involved addition, subtraction, multiplication, or division and required either one or two steps to complete. For example: “A sister has 28 pencils, her brother has 10 pencils. How many pencils does she need to give to her brother so that they each have an equal amount?” One potential solution is to calculate how many more pencils the sister has than her brother (18 pencils) and then divide that difference in half (9 pencils). All the problems were presented randomly and self-paced during 60-s blocks.

### Apparatus and Imaging Parameters

We conducted functional MRI (fMRI) analyses on a Siemens (Munich, Germany) 3T Trio scanner using an 8-channel phase array head coil. Participants laid supine in the scanner with their heads immobilized. A single shot, T2^∗^-weighted gradient-echo echo planar imaging (EPI) sequence was used for the fMRI scans with slice thickness of 6 mm and no gap between slices, in-plane resolution of 3.75 × 3.75 mm, and TR/TE = 3000 ms/30 ms. The field of view was 240 × 240 mm and the acquisition matrix was 64 × 64. Thirty contiguous axial slices parallel to AC-PC were acquired. Three-hundred and sixty-two images were acquired with a total scan time of 724 s in a single run. High-resolution T1-weighted anatomical images were also acquired for each participant (3D, gradient-echo pulse-sequence, TR/TE = 25 ms/6 ms, FOV = 220 mm × 220 mm, 89–92 contiguous slices, matrix = 220 × 220, thickness = 2 mm).

### fMRI Scanning Procedures

The participants were given a practice session outside of the scanner prior to the fMRI analysis. In the scanner, mathematical problems were projected onto the center of a translucent screen and viewed by the participants through a mirror attached to the head coil. The stimuli were presented in black against a white background. The visual angle of each problem was less than 3° in both horizontal and vertical directions.

There was one run for the mathematical problem-solving task and a total of 16 blocks per run: eight blocks for mathematical problem-solving and eight blocks of visual fixation each lasting 30 s. The participants were encouraged to respond as quickly and accurately as possible by using their left or right index finger to press a button. After the participant responded, a new problem was presented after a blank of 1,000 ms. If the participants failed to respond within 20 s, the problem would disappear and a new one was presented. If there were 8 s or less left in a block, instead of presenting a new question, a fixation sign (“+”) was presented for the remainder of the block.

### Neuroimaging Data Preprocessing

Neuroimage preprocessing was performed using SPM12 (Statistical Parametric Mapping, Welcome Department of Cognitive Neurology, London^1^). The functional data set acquired from the experiment consisted of 362 image volumes. Functional images were realigned to the first volume in the scanning session using affine transformations. A mean functional image volume was constructed for each participant from the realigned image volumes. This mean image volume was then used to determine the parameters for spatial normalization; these parameters were then applied to the corresponding functional image volumes for each participant. Spatial smoothing was performed on the normalized functional images using a Gaussian kernel 8 mm full-width at half-maximum (FWHM).

For the block-wise whole-brain analysis, statistical analyses were conducted on the smoothed data using a boxcar design with a canonical hemodynamic response function (HRF). A high-pass filter (186 s) was applied in order to remove low frequency effects and a low-pass filter (4 s) to remove the high frequency noise. The global temporal trend was removed. Contrasts of interests were calculated for each individual (below) and subjected to random effects analyses at the subject-based group level using one-sample *t*-tests.

For all contrasts of brain activation, we used the thresholds from the most lenient *p* < 0.05 uncorrected to *p* < 0.005, *p* < 0.001, to the most stringent *p* < 0.05 corrected with a minimum cluster size of 10 voxels.

## Behavioral and Neuroimaging Results

### Behavioral Results

The 28 participants responded to a total of 1,367 trials (math word problems). About 13.7% of the trials, or 187 of them, were responded to incorrectly. Of the incorrect responses, seven trials involved no keystrokes within the maximum RT of 20,000 ms. The average time for the trials with correct responses (2,726) was 8,064 ms, and the average time for incorrect responses (4,904) was 7,915 ms. There was no significant difference between the two types of trial responses: *F*(1,1365) = 0.372, *p* = 0.542, ${\text{η}}_{\text{p}}^{2}$ = 0.000. Only the trials with correct responses were entered into the RT correlation analysis.

### Brain Activations

**FIGURE 3:**
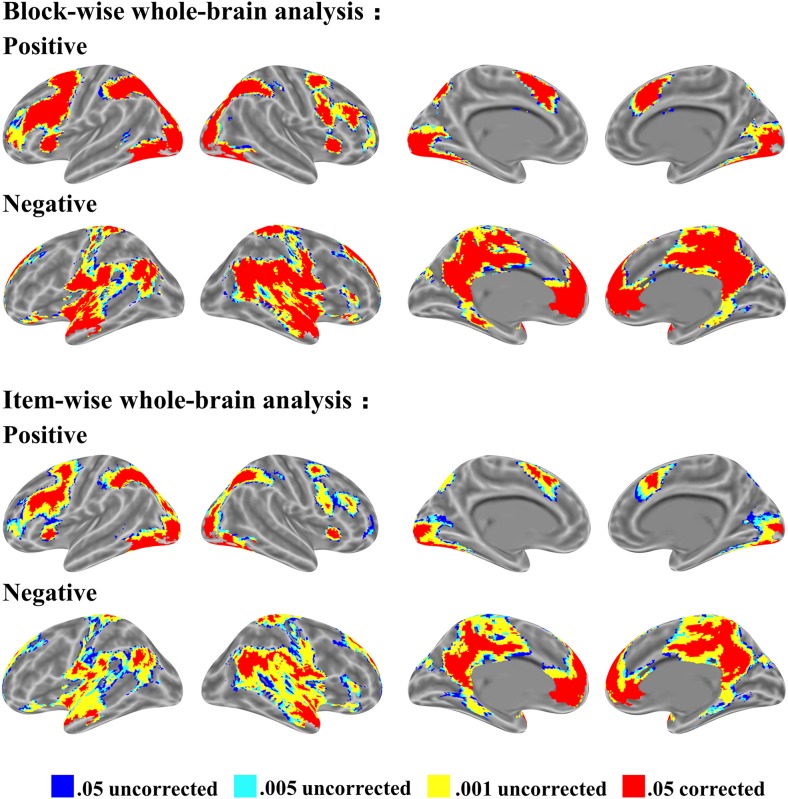
Brain activation for mathematical problem-solving from block-wise subject analysis and item-wise subject analysis. Four thresholds: 0.05 uncorrected, 0.005 uncorrected, 0.001 uncorrected, and 0.05 corrected level each with voxel size > 10, voxel in 3 mm × 3 mm × 3 mm. First row shows positive activation; second row shows negative activation in both block-wise subject analysis and item-wise subject analysis.

The whole-brain activation for mathematical problem-solving is shown in Figure 3. We identified four types of thresholds, 0.05 uncorrected, 0.005 uncorrected, 0.001 uncorrected, and 0.05 corrected, each with more than 10 voxels. Whole-brain activation was observed from two starting points: block-wise analysis and item-wise analysis. The block-wise analysis is the traditional analysis based on block design. The item-wise analysis involves computing the brain activation for each item (i.e., a trial or a math word problem), obtaining a brain map for each participant, and finally, conducting a random effect test. We found that both approaches have similar activation patterns regardless of positive or negative activation. Positive activation is typically located at the bilateral occipital, parietal, and frontal cortex. Negative activation is located at the default mode network including the angular gyrus, anterior temporal cortex, precuneus, and medial prefrontal cortex.

## Item-Wise and Mean-Wise Correlation for Mathematical Problem-Solving

We used a Lilliefors test to determine whether our data (β value) satisfies normal distribution. The Lilliefors test is a normality test based on the Kolmogorov–Smirnov test (Lilliefors, 1969). We tested the activation (β value) of each voxel for all participants to find that 82.2% voxels satisfy normal distribution. The RT of each trial for all participants was also tested: 88.3% trials satisfy normal distribution. Therefore, the Pearson correlation method is applicable.

For the fMRI study wherein 28 participants finished 60 trials, the mean-wise correlation was computed on the 28 mean values of behavioral measure (i.e., RT) to obtain 28 brain activation maps with each voxel of each map involving the mean of brain activation based on the 60 trials. This process results in a *t* map where each brain voxel has a *t-*value.

**FIGURE 4:**
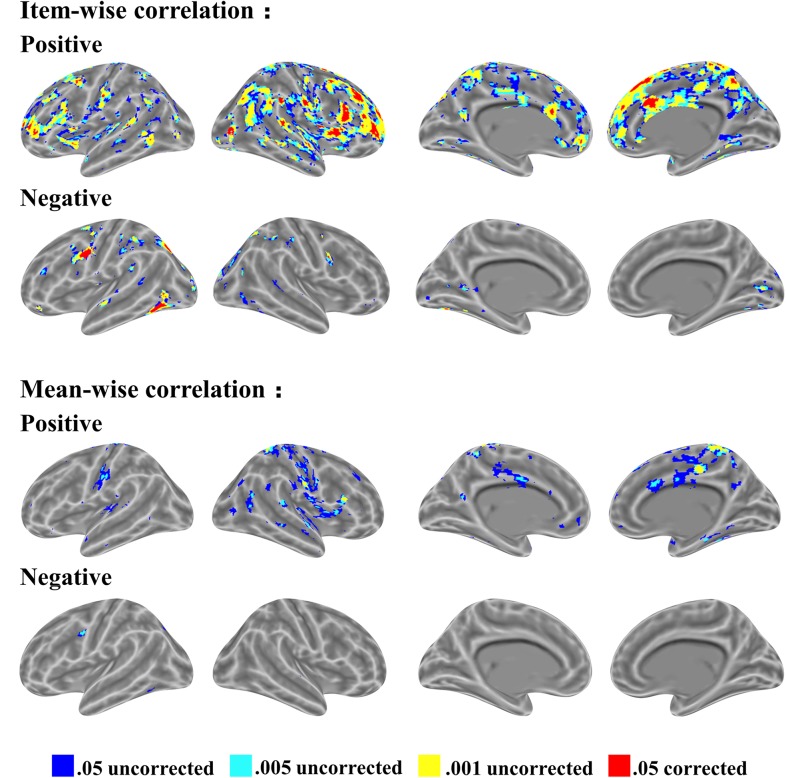
Significant clusters of item-wise and mean-wise interindividual behavior and brain correlation with voxel size > 10 (voxel in 3 mm × 3 mm × 3 mm).

In the item-wise correlation, we first computed the correlation between the RT of 28 participants and their brain maps for an item (resulting in 60 *r-*values for each brain voxel) and conducted a one-sample *t-*test on the 60 *r-*values. This analysis also results in a *t* map. The *t* maps for item-wise correlation and mean-wise correlation between brain activation (i.e., β value) and RT are shown in Figure 4, again with four thresholds: 0.05 uncorrected, 0.005 uncorrected, 0.001 uncorrected, and 0.05 corrected. Table 1 reports the brain regions with positive and negative brain-behavior correlations based on item-wise correlation analysis via Pearson method (Figure 4).

**Table 1:** Brain regions with positive and negative brain-and-behavior correlations based on item-wise correlation at p < 0.05 corrected (voxel size > 10).

Brain region	Volume	*t-*Value		Coordinates	
**Item-wise positive correlation**					
Right middle occipital gyrus	23	10.01	45	-81	9
Left middle frontal gyrus	39	9.98	-33	6	63
		5.31	-33	3	51
Right middle frontal gyrus	146	9.15	39	57	9
		6.63	24	54	21
		5.86	27	63	12
Left inferior frontal gyrus (Operculum)	39	8.52	-39	15	18
		6.43	-36	12	-9
		5.83	-39	12	0
Right inferior frontal gyrus (Triangle)	223	8.25	27	27	30
		7.56	33	15	12
		7.10	54	24	18
Right superior temporal gyrus	11	8.23	66	-18	9
Right medial superior frontal gyrus	214	8.02	3	33	51
		7.75	9	21	30
		7.33	0	27	39
Right precentral gyrus	33	7.91	63	-3	30
Left middle frontal gyrus	75	7.52	-30	57	9
		6.08	-36	45	6
		5.83	-45	51	3
Right superior temporal gyrus	18	6.94	69	-36	12
		5.81	69	-27	3
Right superior frontal gyrus	56	6.77	18	9	66
		6.36	27	3	66
		6.03	36	9	63
Left superior frontal gyrus (Orbital)	12	6.76	-15	48	-9
Right supramarginal gyrus	12	6.63	63	-33	39
Left superior parietal lobule	14	6.52	-27	-81	45
Left precuneus	12	6.45	-9	-60	66
		5.92	-15	-66	66
Left supplementary motor area	13	6.40	-12	6	60
		5.81	-12	12	66
		5.66	-12	21	63
Right superior frontal gyrus	10	6.08	21	21	60
Right precuneus	17	6.07	6	-45	69
Right postcentral gyrus	10	6.06	24	-42	69
Right middle frontal gyrus	10	5.78	39	39	21
**Item-wise negative correlation**					
Left precentral gyrus	48	-9.10	-51	0	39
Left inferior temporal gyrus	62	-7.64	-42	-57	-6
		-6.62	-30	-72	-9
Left superior occipital gyrus	41	-7.11	-21	-60	36
Left inferior parietal lobule	10	-6.19	-48	-36	51

We observed positive correlations mostly at the SMA, right middle temporal gyrus, bilateral amygdala, medial superior frontal gyrus, bilateral fusiform gyrus, and bilateral superior temporal gyrus under the threshold *p* < 0.05 corrected. Negative correlations are generally located at the bilateral thalamus, bilateral cerebellum, bilateral inferior temporal gyrus extending to the inferior occipital gyrus and fusiform, bilateral middle frontal gyrus, left inferior frontal gyrus, and right postcentral gyrus under the threshold *p* < 0.05 corrected.

Table 2 shows the brain regions with positive and negative brain-behavior correlations based on traditional mean-wise correlation analysis (Figure 4). We found positive correlations mostly at the bilateral postcentral gyrus, right precentral gyrus, bilateral superior temporal gyrus, middle cingulum gyrus, supplementary motor area, and right fusiform gyrus under the threshold *p* < 0.05 uncorrected. Negative correlations were observed at the left precentral gyrus, left inferior parietal lobule, left superior occipital gyrus, and left inferior occipital gyrus extending to the inferior temporal gyrus under the threshold *p* < 0.05 uncorrected.

**Table 2:** Brain regions with positive and negative brain-and-behavior correlations based on mean-wise correlation at p < 0.05 uncorrected (voxel size > 10).

Brain region	Volume	*t-*Value		Coordinates	
**Mean-wise positive correlation**					
Right postcentral gyrus	3061	6.29	24	-45	66
		5.14	60	-6	30
		5.12	-12	-42	75
Right cerebellum (6)	58	4.71	18	-60	-33
Left cerebellum (9)	24	4.40	-15	-48	-39
Left inferior frontal gyrus (Orbital)	14	4.32	-30	33	-18
Left precentral gyrus	30	4.30	-36	-24	66
Left insula	15	4.01	-36	12	18
Left precuneus	60	3.75	-12	-48	60
Left superior temporal gyrus	136	3.60	-45	-30	12
		2.83	-66	-39	15
		2.74	-42	-15	-3
Left postcentral gyrus	151	3.57	-51	-15	33
		2.35	-66	-9	21
Right middle temporal gyrus	192	3.56	45	-60	12
		2.79	39	-75	39
		2.62	57	-60	30
Left calcarine	51	3.39	-6	-60	9
		2.50	3	-42	12
Right medial superior frontal gyrus	48	3.36	9	36	51
Left superior temporal pole gyrus	19	3.25	-45	24	-15
Right middle temporal gyrus	25	3.25	54	-36	-9
Left middle frontal gyrus	31	3.22	-33	60	12
Left inferior frontal gyrus (Triangle)	58	3.22	27	27	30
Left superior temporal pole gyrus	15	3.19	-36	18	-30
Left cerebellum (4–5)	48	3.18	-27	-39	-27
		2.48	-27	-57	-36
Left superior occipital gyrus	18	3.08	-27	-84	42
Left anterior cingulum	65	3.07	0	24	-3
		2.58	0	54	-12
Right precuneus	24	3.06	6	-54	24
Left cerebellum (6)	14	2.98	-33	-63	-27
Left middle temporal gyrus	13	2.93	-54	6	-21
Right hippocampus gyrus	13	2.87	15	-36	12
Right medial superior frontal gyrus	39	2.83	9	57	21
Left superior frontal gyrus	12	2.81	-24	-6	69
Left thalamus	22	2.80	0	-21	18
Left superior temporal gyrus	30	2.80	-45	6	-9
Left middle frontal gyrus	13	2.80	36	57	9
Left cerebellum (4–5)	13	2.77	-6	-54	-6
Vermis (10)	20	2.76	0	-36	-36
Left thalamus	13	2.70	-3	-18	-9
Right cerebellum (6)	12	2.65	27	-75	-18
Left anterior cingulum	15	2.64	-12	48	-3
Left amygdala	11	2.39	-30	0	-12
**Mean-wise negative correlation**					
Left precentral gyrus	35	-3.53	-48	-3	39
Left inferior parietal lobule	22	-2.91	-45	-42	48
Left inferior temporal gyrus	28	-2.81	-39	-54	-3
Left superior occipital gyrus	13	-2.54	-21	-60	33

There were several brain regions that showed similar significant correlations both item-wise and mean-wise. The brain regions containing positive correlations include the bilateral precuneus, bilateral superior frontal gyrus, right postcentral gyrus, and left middle frontal gyrus; the same brain regions that showed negative correlations include the left precentral gyrus, left inferior parietal lobule, and left inferior temporal gyrus.

**FIGURE 5:**
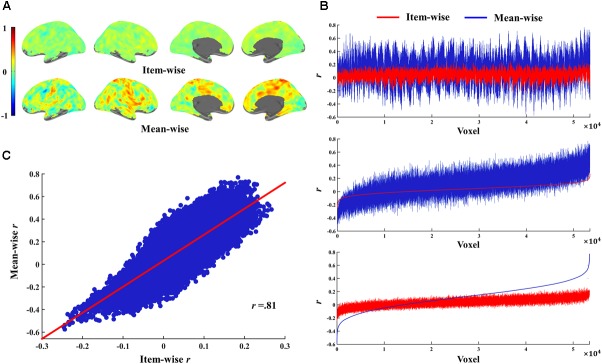
Relations of r-values for item-wise and mean-wise brain-behavior interindividual correlation and their correlation. **(A)** r-Value maps for item-wise and mean-wise correlation. **(B)** Relation of r-values at voxel level for the two correlation. Top: voxels ordered by coordinates. Middle: voxels ordered by item-wise r-values. Bottom: voxels ordered by mean-wise r-values. **(C)** Scatter map of r-values for two correlations.

The relations of *r-*values in the two correlations are shown in Figure 5; the brain maps for the same *r-*values are shown in Figure 5A. We found that the *r-*value from item-wise correlation is generally smaller than that from mean-wise correlation (Figure 5B). The correlation between the *r-*values from the item-wise correlation and the *r-*values from the mean-wise correlation across all voxels in the whole brain results in a value of 0.81 (Figure 5C). We also found that 92.7% voxels (β value) positively or negatively correlated with RT under the threshold of *p* < 0.05 uncorrected by mean-wise correlation were observable via item-wise correlation analysis. Only 19.8% voxels (β value) positively or negatively correlated with RT under the threshold of *p* < 0.05 uncorrected by item-wise correlation analysis were observable via mean-wise correlation. That is, 80.2% of the voxels with significant correlation by item-wise correlation analysis were not observable by mean-wise correlation analysis (Figure 4). This suggests that item-wise correlation analysis reveals more significant correlations than mean-wise.

## Cross Validation

**FIGURE 6:**
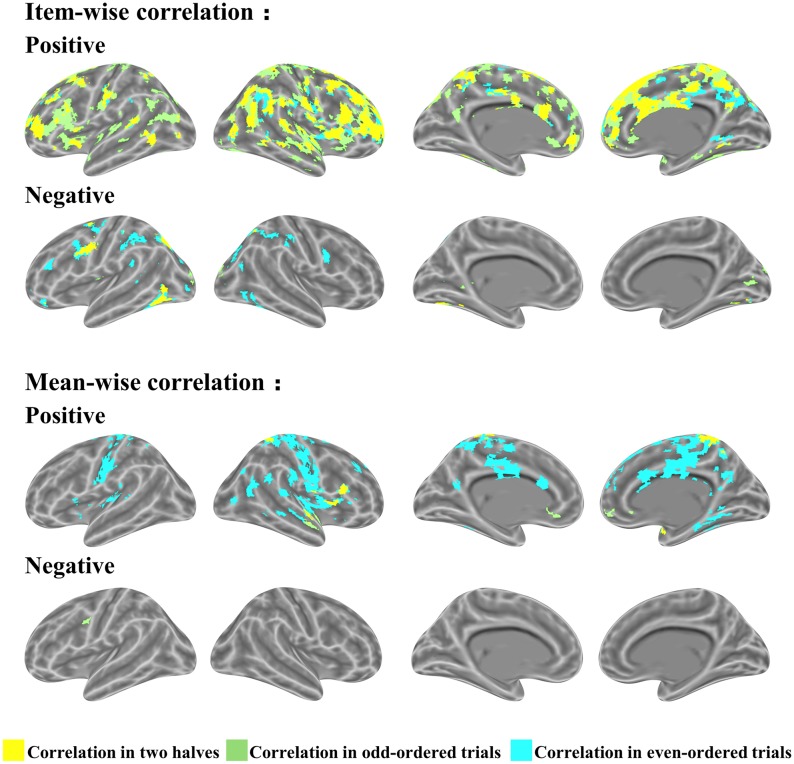
Cross validation analysis on odd- and even-ordered trials (p < 0.05 uncorrected for both item-wise and mean-wise correlations).

We ran a cross validation to assess the consistency of all the items contributing to the association between brain and behavior. The items were split into two halves according to their parity: odd-ordered trials and even-ordered trials. The item-wise correlation led to high consistency between the two trial halves (Figure 6). The same brain regions activated in the two correlations (odd- and even-ordered trials) is marked in yellow in the figures below. Table 3 reports the same brain regions with positive or negative brain-behavior correlation for odd- and even-ordered trials. The clusters of activation in the odd-ordered trials with *p* < 0.05 corrected were repeated in the clusters of activation in even-ordered trials.

**Table 3:** Same brain regions with positive or negative brain-behavior correlations for odd- and even-ordered trials (*p* < 0.05 uncorrected, voxel size > 10).

Brain region	Volume	*t-*Value		Coordinates	

**Item-wise positive correlation**					

Right middle occipital gyrus	1009	7.07	45	-81	9

		5.81	66	-18	9
		5.73	63	-3	30
Left middle frontal gyrus	108	7.03	-33	6	63
		3.22	-24	12	45

Right middle frontal gyrus	3510	6.68	39	57	9

		6.24	-39	15	18
		5.82	30	21	27

Left middle occipital gyrus	139	4.81	-27	-81	45

		3.27	-9	-87	36
		3.00	6	-60	24

Left superior frontal gyrus (Orbital)	67	4.79	-15	48	-9

Vermis (3)	46	4.72	6	-36	-6

Right precuneus	539	4.55	9	-57	48

		4.52	-9	-60	66
		4.45	6	-42	69
Right supplementary motor area	37	4.36	9	-3	69
Left inferior temporal gyrus	61	4.00	-57	-63	-3
		2.84	-42	-87	3

Left middle cingulum	98	3.98	0	-18	18

		2.95	-3	-3	36

Left postcentral gyrus	73	3.92	-51	-15	36

Left cerebellum (6)	31	3.76	-27	-48	-21

Left middle temporal gyrus	38	3.72	-63	-36	12

Left cerebellum (6)	79	3.66	-33	-63	-27

		3.04	-30	-78	-45

Left thalamus	18	3.63	-3	-18	-9

Right fusiform gyrus	28	3.50	24	-48	-12

Left caudate	22	3.50	-15	-12	24

Right precentral gyrus	32	3.46	18	-21	66

Left superior frontal gyrus	14	3.32	-15	45	42

Right precentral gyrus	42	3.25	39	-12	48

Left middle occipital gyrus	12	2.98	-42	-84	18

**Item-wise negative correlation**					

Left precentral gyrus	133	-6.72	-51	0	39

		-3.59	-36	-9	21

Left fusiform gyrus	160	-5.39	-42	-57	-6

Left middle occipital gyrus	117	-5.01	-21	-60	36

		-3.33	-18	-75	54

Right fusiform gyrus	48	-4.43	33	-66	-6

Left postcentral gyrus	52	-4.35	-48	-36	51

Left middle frontal gyrus (Orbital)	11	-4.27	-30	42	-6

**Mean-wise positive correlation**					

Right postcentral gyrus	181	6.47	24	-45	66

		3.04	9	-54	51

Left precuneus	69	4.79	-12	-42	75

		3.11	3	-27	60

Right hippocampus gyrus	152	3.78	18	-3	-9

		3.43	48	18	-12

Right superior temporal gyrus	75	3.74	57	-3	6

Right inferior frontal gyrus (Triangle)	71	3.57	48	24	18

		3.49	36	6	9
Mean-wise negative correlation					
None					

*Consistent brain regions identified by overlaying and averaging odd- and even-ordered trials with correlation at *p* < 0.05 uncorrected*.

**FIGURE 7:**
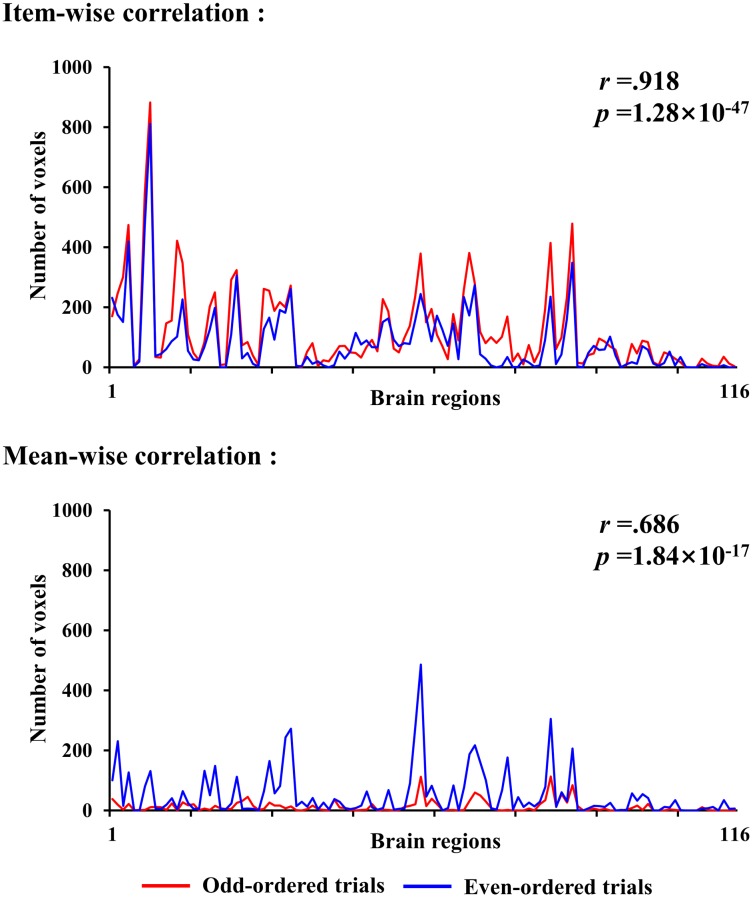
Number of voxels for odd- and even-ordered trials in AAL template (116 brain regions) for item-wise and mean-wise correlations (p < 0.05 uncorrected for both item-wise and mean-wise correlations).

The AAL atlas is used here to show the statistical stability at brain region level. The correlation of activated voxel quantities in 116 regions of the AAL template is 0.918 and the mean-wise correlation is only 0.686 (Figure 7). The *p-*value is higher and *r-*value is lower in the mean-wise correlation than the item-wise, which suggests that mean-wise correlation analysis is less stable.

## Relation Between Interindividual and Intraindividual Approaches

Interindividual brain-behavior correlation tends to reflect the variations associated with individuals, but intraindividual brain-behavior correlation tends to reflect the variations associated with items. Both inter- and intraindividual correlations reflect variations in brain activation related to cognitive processing. An example for each approach is provided in Introduction section.

**FIGURE 8:**
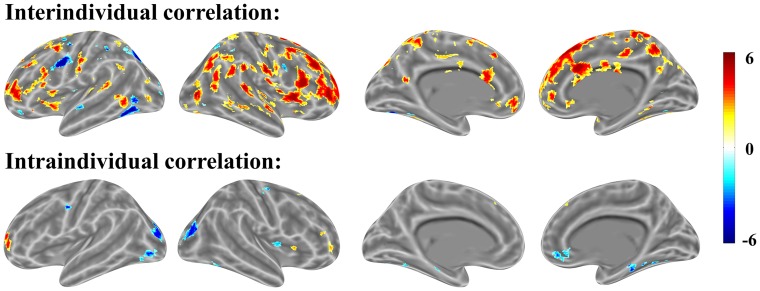
Interindividual (top) and intraindividual (bottom) correlations in mathematical problem-solving (p < 0.001 uncorrected for both interindividual and intraindividual correlations).

In our mathematical problem-solving experiment, we first performed intraindividual brain-behavior correlation for each individual across items/trials, then combined the correlation brain maps with single-sample *t-*tests against 0 (Figure 8). The intraindividual map and interindividual map was correlated with 0.306 according to the *t-*value at the voxel level for mathematical problem-solving. According to Cohen’s rules of thumb (1988, 1992), *r* in 0.306 is “medium” level; only 10% of the variation can be explained. The result suggests that the two correlations are independent.

We found that interindividual correlation is altogether more practical than intraindividual correlation. The ideal experimental design involves keeping the trials homogeneous, which greatly reduces the brain-behavior correlation. Conversely, recruiting participants across the widest possible range creates better sample distribution and more meaningful across-subject correlation.

## Relation Between Brain Activation and Correlation

**FIGURE 9:**
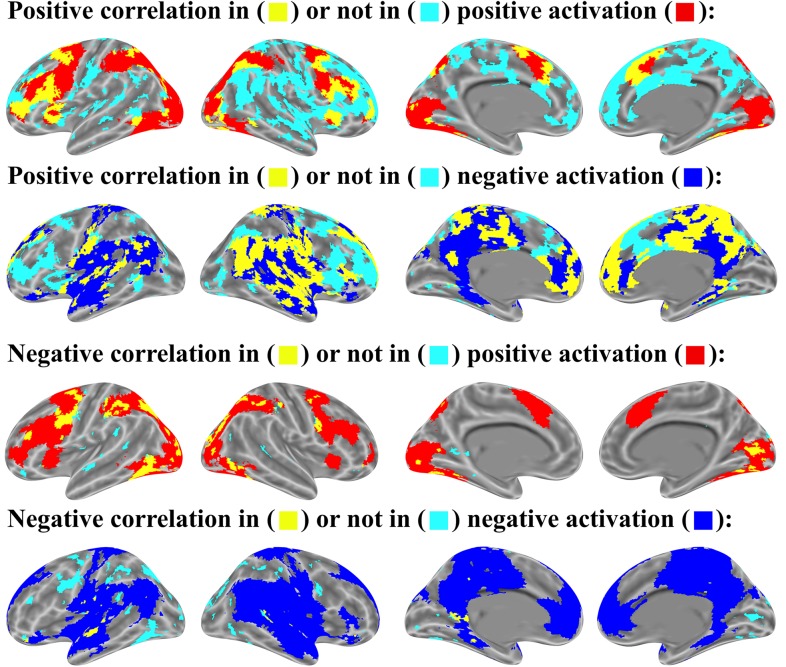
Item-wise correlation in brain activation map (p < 0.05 uncorrected for both correlation and activation analysis, number of activated voxels exceeds 10).

**FIGURE 10:**
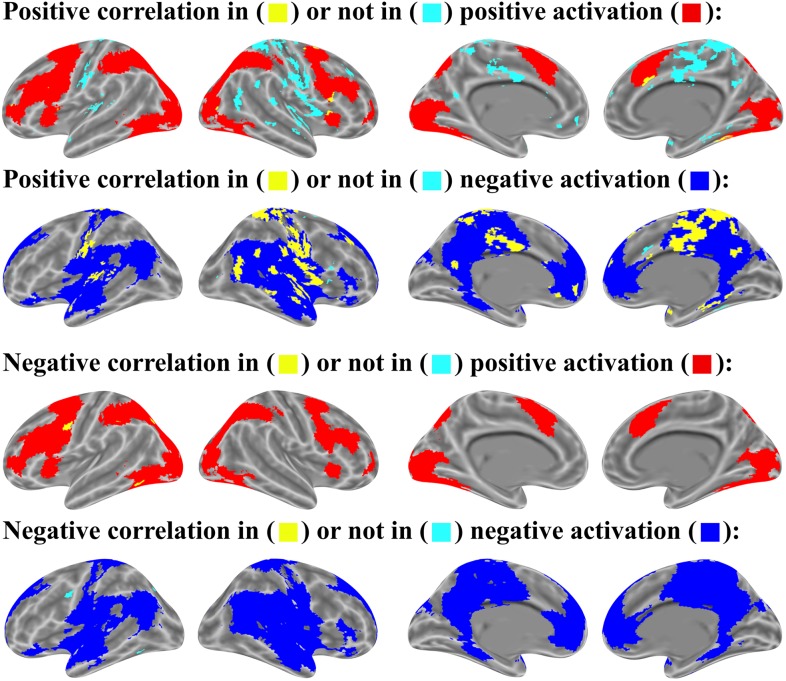
Mean-wise correlation in brain activation map (p < 0.05 uncorrected for both correlation and activation analysis, number of activated voxels exceeds 10).

To determine the proportion of voxels with significant correlations in the positively or negatively activated brain voxels (see section “Brain Activations”), we set the most lenient threshold (*p* < .05 uncorrected) to cover the widest possible range of activated and associated brain regions. The relation is shown in Figures 9, 10.

For the relation between brain activation and item-wise correlation, about 25.8% of brain voxels with the positive correlation were found in the positively activated brain and 51.3% in the negatively activated brain. About 76.3% of brain voxels with negative correlation were observed in the positively activated brain, but only 7.4% in the negatively activated brain. (Figure 9). For the relation between brain activation and mean-wise correlation, about 8.0% voxels that showed positive correlation were displayed in the positively activated brain and 74.5% in the negatively activated brain. About 96.9% voxels that showed negative correlation were observed in the positively activated brain, and no voxel was observed in the negatively activated brain (Figure 10).

In addition, our item-wise correlation analysis showed that the correlation of some voxels meets the threshold of *p* < .05 corrected. The relation between brain activation and the item-wise correlation was computed with the threshold of *p* < .05 corrected. Up to 33.9% of the brain voxels with positive correlation were observed in the positively activated brain and 27.0% in the negatively activated brain. Up to 98.8% of brain voxels with negative correlation were found in the positively activated brain, but no voxels were observed in the negatively activated brain.

According to the results of item-wise and mean-wise correlation analyses, negative correlation between brain activation and RT is generally found in the brain regions with positive activation (see section “Brain Activations,” “whole-brain activation analysis”). Positive correlation can be found in both positively and negatively activation brains.

## Discussion

The goal of this study was to secure a novel, simple correlation procedure for brain-behavior association analysis across individuals and ultimately to shed light on how the brain processes information, as well as how individuals differ from one another in regards to cognition.

Traditional mean-wise individual brain-behavior correlation involves the pursuit of the highest possible correlation. Behavioral measurement techniques are limited, so the correlation cannot be especially high. Item-wise individual brain-behavior correlation, conversely, centers around the pursuit of weak but stable correlation coefficients. The weak correlation for an item arises due to an abundance of noise.

The statistical power in simulations on data-containing signals is enormously enhanced by this technique. Comparison between the two correlation approaches on any given example (e.g., mathematical problem-solving) also shows that only the item-wise correlation can meet the gold standard for activation detection. Cross validation also reveals stable correlation patterns under the item-wise correlation approach. The removal of noise during the correlation analysis of each item is why the item-wise correlation is so successful.

The linear regression model can be borrowed to demonstrate the working principle of item-wise correlation. The residual error (*𝜀_i_*) can be filtered out in the model. The regression coefficient in a simple linear regression can directly be transformed into the correlation coefficient *r* = *β*^∗^*SD_x_*/*SD_y_*. The mean-wise correction on random data (without any signal) results in larger absolute *r-*value, which indicates more false alarm error (type I error).

Enhanced statistical power lends strength to the application of correction for multiple testing in correlation analysis, which is otherwise a serious problem (Rousselet and Pernet, 2012). The present study was conducted to test the effectiveness of item-wise interindividual brain-behavior correlation for task fMRI studies. An fMRI experiment on mathematical problem-solving was run and analyzed with a typical item-wise correlation process. Task fMRI studies actually are a subset of neuroimaging studies – the question of whether item-wise correlation applies to other types of neuroimaging studies (e.g., on data collected outside the scanner) remains to be answered. We would assert that item-wise correlation analysis is indeed valid for studies with behavioral data collected outside the scanner. If there is a one-to-one corresponding relation between trials outside the scanner and inside (i.e., data can be paired), the item-wise analysis applies across the board. Behavioral data was collected outside an fMRI scanner (e.g., reaction time for 28 trials) and brain activation was collected as the participants completed 60 trials inside the fMRI scanner. There were no one-to-one corresponding relations between the sets of trials. The data (e.g., RT) for each trial collected outside the scanner can also correlate the brain activations of each trial inside the scanner. Average data of all trials for each participant collected outside the scanner can also correlate with the brain activations in each trial.

In a resting fMRI study, behavioral data is collected outside the scanner or collected in a secondary session inside the scanner. The behavioral data for each trial can be correlated with brain signals in the resting state. The resting state can also be separated into different time windows, e.g., with 15 to 30 TRs per window (Handwerker et al., 2012; Hutchison et al., 2013; Leonardi et al., 2013; Allen et al., 2014). The average data of all trials for each participant can be correlated with the brain signal for each time window in the resting state.

It should be noted that the proposed model (i.e., item-wise individual brain-behavior correlation) is actually a random-effects model on the items, and a fixed-effects model on subjects. The effect from statistical test can be generalized to new items from the same subjects. On the other hand, it is hard to make a prediction for new subjects that would be appended to the cohort.

## Conclusion

The paper introduced a novel brain-behavior correlation method with markedly enhanced statistical power. The proposed method may effectively resolve problems inherent to traditional brain-behavior correlation techniques. Cross validation demonstrated the reliability of the item-wise interindividual correlation, which was also applied to a block-design neuroimaging study. The analysis technique discussed here could be extended to event-related design in future neuroimaging studies, as it can be used to conveniently and accurately model brain activation across multiple trials.

## Ethics Statement

This study was carried out in accordance with the recommendations of guidelines from the Institutional Review Borad of BNU Imaging Center for Brain Research, State Key Laboratory of Cognitive Neuroscience And Learning. The protocol was approved by the Institutional Review Borad of BNU Imaging Center for Brain Research, State Key Laboratory of Cognitive Neuroscience and Learning.

## Author Contributions

XZ proposed research questions and hypotheses, performed the data analyses, and wrote the manuscript. ML performed the data analyses and wrote the manuscript. HZ, LL, and JC performed the data analyses.

## Conflict of Interest Statement

The authors declare that the research was conducted in the absence of any commercial or financial relationships that could be construed as a potential conflict of interest.

## Footnotes

1 http://www.fil.ion.ucl.ac.uk/spm/
